# Evolutionary and Medical Consequences of Archaic Introgression into Modern Human Genomes

**DOI:** 10.3390/genes9070358

**Published:** 2018-07-18

**Authors:** Olga Dolgova, Oscar Lao

**Affiliations:** Population Genomics Group, Centre Nacional d’Anàlisi Genòmica, Centre de Regulació Genòmica (CRG-CNAG), Parc Científic de Barcelona, Baldiri Reixac 4, 08028 Barcelona, Catalonia, Spain

**Keywords:** archaic introgression, fitness, natural selection, Neanderthal, Denisovan, anatomically modern humans

## Abstract

The demographic history of anatomically modern humans (AMH) involves multiple migration events, population extinctions and genetic adaptations. As genome-wide data from complete genome sequencing becomes increasingly abundant and available even from extinct hominins, new insights of the evolutionary history of our species are discovered. It is currently known that AMH interbred with archaic hominins once they left the African continent. Modern non-African human genomes carry fragments of archaic origin. This review focuses on the fitness consequences of archaic interbreeding in current human populations. We discuss new insights and challenges that researchers face when interpreting the potential impact of introgression on fitness and testing hypotheses about the role of selection within the context of health and disease.

## 1. Widespread Interbreeding between Hominins

**Figure 1:**
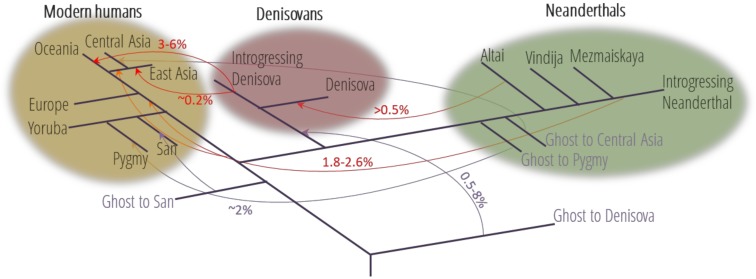
Family tree of the four groups of early humans living in Eurasia 50,000 years ago and the inferred gene flow between the groups due to interbreeding (based on [12,13,14,15,16]). The direction and estimated magnitude of inferred gene flow events are shown. Branch lengths and timing of gene flows are not scaled. Light violet color indicates introgression events from unknown archaic populations (Ghost).

The demographic history of anatomically modern humans (AMH) is complex, and involves a large number of migrations, genetic admixtures and introgressions, population extinctions and genetic adaptations, which overlap both in time and in space (see Figure 1). Due to this complexity, the evolutionary history of humankind is still far from being fully understood [1]. During the last 30 years, the most accepted demographic scenario for explaining recent evolution of AMH has been the Out of Africa model (OOA). According to this model, AMH evolved in Africa around 100–200 thousand years ago (kya) in East Africa [2,3] and migrated to the rest of the world around 50–60 kya [4,5,6,7]. This widely accepted dating of *Homo sapiens* emergence was recently challenged by Hublin et al. [8], who found AMH fossils of 300 kya at Jebel Irhoud in Morocco [9]. Similarly, Herschkovitz et al. [10] described a *H. sapiens* maxilla of 177 to 194 kya in Misliya Cave, Israel. All these studies suggest that the members of the *H. sapiens* clade left Africa earlier than previously thought, probably in several waves of OOA migration at different stages of evolution. Classical “pure” OOA assumes that admixture with other archaic populations such as Neanderthals or Denisovans, present at the time of the rise of AMH, either did not occur or was negligible [11].

However, genomic studies of ancient DNA have revealed that AMH interbred with other hominid lineages, such as Neanderthals and Denisovans, present in Eurasia since 300 kya up to 30–50 kya. The admixture with Neanderthals occurred around 37–86 kya, and most likely between 47 and 65 kya [17,18,19,20]. The event of admixture with Denisovan took place within similar time span, ~44–54 kya [21]. Whole-genome sequences from ancient specimens [22] have revealed that Non-African populations outside Oceania carry between 1.8% and 2.6% of Neanderthal DNA (Figure 1) [13]. As described in [18], East Asians carry somewhat more Neanderthal DNA (2.3% to 2.6%) than people in Western Eurasia (1.8% to 2.4%). In contrast, DNA introgressed into modern humans from Denisovans is found mostly in Australo-Melanesians, which may account for up to 6% of Denisovan DNA in their genomes and, to a lesser extent, in South Asians [23] and East Asians [24]. These estimates are averages across the modern human genome. However, specific regions of the genome may have degrees of Neanderthal ancestry as high as 64% in Europeans and 62% in Asians.

New studies based on current genetic diversity are suggesting that the events of archaic introgression in AMH did occur after out of Africa migration with other hidden ”ghost” archaic populations [14]. The main difficulty for the inference of archaic introgression in African modern human genomes from African archaic populations is mainly due to the current absence of genetic material from the remains of archaic hominins that could be used as a proxy for studying the source of introgression, as the climate of African continent is not favorable for DNA preservation. Nevertheless, there is growing evidence that archaic introgression occurred also within this continent [15,25,26,27,28,29], raising the exciting possibility that other unknown archaic groups may have contributed to human genetic diversity. Therefore, recent work suggests that apparently distinct species can exchange the genetic material along their evolutionary history [30]. The biological implications of such introgression, including their consequences on modern human health, is reviewed in the following sections.

## 2. Selection against Introgressed Regions at the Level of Genomes and Individual Loci

Introgressed alleles in a foreign genetic background frequently have negative fitness effects regardless of the amount of adaptive introgression. Martin and Jiggins [31] made two important considerations when dealing with models of selection against introgressed genomic tracts. First, as many of the factors that influence selection—such as recombination rate and gene density—are interdependent, the models that account for combined effects of both factors are more feasible, especially if they incorporate specific predictions such as the decline in selective sweep strength with increasing distance from selected loci. Juric et al. [32] modelled the level of Neanderthal ancestry in human populations as a function of the recombination distance from nearby selected alleles and estimated both the density of selected loci and the strength of selection. Second, interpretations of the landscape of ancient introgression into human species may vary depending on underlining assumptions. For example, the majority of models assume that introgressed blocks are selected independently of each other in the genetic background of the recipient population. However, Harris and Nielsen [33] showed that much of the selection against introgression may occur in early generations, since early generation hybrids should have complex ancestries in which epistasis can lead to non-additive fitness effects. Another assumption pointed out in this study is that weakly deleterious mutations segregating in the donor population would be the main driver of selection against Neanderthal introgression in humans. Under such a model, the lower effective population size in Neanderthals would have led to the accumulation of weakly deleterious alleles that, once introgressed into humans, would reduce the relative fitness of the hybrid. However, in such context, even if both species bear recessive deleterious alleles but at differing sites, hybrids might have enhanced overdominant fitness variation regardless these deleterious recessives, which leads to the conclusion that Neanderthal introgression may have initially been favored by selection in humans [33].

Disproportionate roles for sex chromosomes in species differences and hybrid incompatibility constitutes a consistent pattern in speciation [34,35]. The compelling evidence of these processes has been reported in the genomes of non-African humans, which have sequences devoid of introgressed variation (“deserts”) from Neanderthals and Denisovans, possibly driven by selection against introgression described by Sankararaman et al. [21,36]. Furthermore, the authors indicated that the introgression deserts of Neanderthal and Denisovan DNA in modern humans are largely overlapping. Of particular interest is a significant reduction in admixture associated with genes showing testes-specific expression, suggesting that admixture may have led to reduced male fertility and supporting evidence of reduced introgression on sex chromosomes [21,36,37]. However, this genomic evidence must be interpreted with caution [38]. When selection against introgression occurs at a large number of loci throughout the genome, its combined effects on many loci can leave detectable patterns, even though selection on any individual locus may be weak [31]. Moreover, weaker signals of introgression have been observed in parts of the genome with high gene density and/or low recombination [21], agreeing with theoretical work, which predicted that the strength of selection against introgression depends on the density of selected sites and the recombination rate [39].

**Figure 2:**
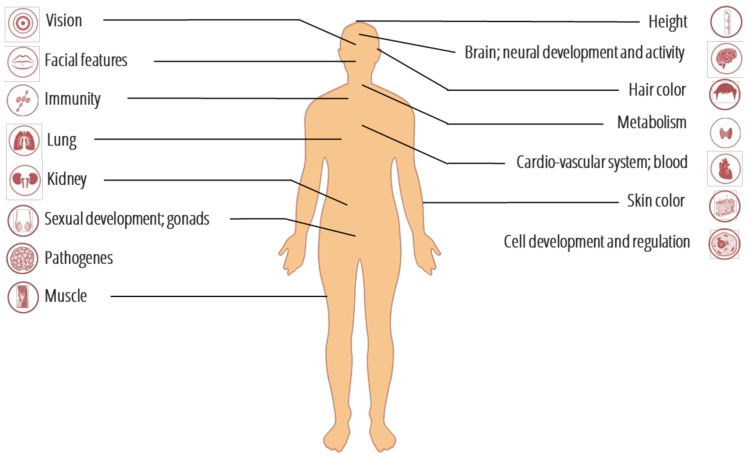
The modern human organs and systems affected by introgressed variants from ancient genomes (see Table S1 for details).

Evidence for the role of purifying selection in shaping the introgression landscape comes from particular categories of genes experiencing different amounts of introgression as previously demonstrated for non-human species [40,41,42,43]. This is also true for the autosomal regions deficient in both Neanderthal and Denisovan ancestries, which contain a significant enrichment of genes transcribed in meiotic germ cells [44,45]. The phenotypic traits affected by archaic introgression are summarized in Figure 2, and their corresponding genomic regions with the type of selective regime acting on them are listed in the Table S1. Taking into account that there has been strong selection against archaic introgression among protein-coding genes [21,46,47], functional regions contributing to the uniqueness of some modern human traits could be identified if they are strongly depleted of archaic ancestry [48]. For example, no Neanderthal ancestry has been detected around the forkhead box protein P2 (*FOXP2*) gene [21], mutations of which are associated with language disorders [49]. Similarly, Neanderthals and Denisovans carry a single copy of *AMY1* gene, encoding an amylase enzyme responsible for starch digestion [21]. In contrast, AMH carry multiple copies of the gene [50,51] and there is no evidence of Neanderthal introgression [21]. This has been interpreted as an evidence that the production of larger amounts of salivary amylase for starch digestion has been under positive selection in modern humans compared to archaic species [52]. Moreover, regions depleted of both Neanderthal and Denisova ancestry are enriched for genes expressed in specific brain regions (e.g., the ventral frontal cortex-ventrolateral prefrontal cortex in infants and the striatum in adulthood; [48]). Another genomic study of Chintalapati et al. [53] on small indels introgressed from Neanderthal demonstrated that negative selection affected these variants more than other variants segregating in modern humans and confirmed that deletions evolved under more constraint than insertions, the vast majority of them laying in the intronic regions. Besides, introgressed variants that may influence on the phenotype of their carriers were identified (Table S1). Among them, an introgressed deletion associated with a decrease in the time to menarche may constitute an example of a former Neanderthal-specific trait contributing to modern human phenotypic diversity [53] (Table S1).

Further evidence of the deleterious effect of Neanderthal introgression can be identified at the expression level. Analysis of gene expression of Neanderthal alleles in current individuals shows a significant downregulation in the testes and brain compared to other tissues [54,55].

## 3. Genomic Signatures of Adaptive Introgression from Archaic to Modern Humans

The footprint of purifying selection against archaic alleles is widespread in the human genome. Nevertheless, given that archaic species evolved for long times in environments for which early AMH were not biologically adapted, interbreeding between anatomically modern humans with archaic species could have facilitated adaptation to specific environments [56,57] (see Table S1). This evolutionary process could bring variants at a higher frequency than de novo mutations, providing linked blocks of sequence with multiple functional mutations, potentially including co-adapted alleles [58]. This process, known as adaptive introgression, has risen to prominence based on a series of high profile examples in human genomes [56,59,60] (see Figure 2). For example, genes involved in functions related to keratin filaments, sugar metabolism, muscle contraction, body fat distribution, enamel thickness, oocyte meiosis, brain size and functioning have been targeted by adaptive introgression from Neanderthals in different non-African genomes [29,36,56,61,62,63,64,65]. Genes involved in the variation of skin pigmentation and hair morphology (*BNC2, MC1R*) also show the signature of positive selection as the result of adaptation to diverse habitats with different degree of insolation (Table S1) [54,66,67]. Advantageous immune variants introduced into the modern human population from archaic genomes have substantially contributed in the present-day diversity of immune genes [56,68,69,70,71,72]. Since innate immunity genes have evolved under stronger purifying selection than the rest of the genome [73], this enrichment of introgressed alleles suggests the presence of strong positive selection at the immune system. A broader overview on ancient pathogens transmitted into modern human genomes through sexual interactions with archaic hominins and their impact on AMH immunity evolution can be found in the review of Pimenoff et al. [74] published in this Special Issue on Evolutionary Medicine. Similarly to innate immunity genes, *EGLN1* and *EPAS1* genes, associated with hemoglobin concentration and response to hypoxia, display a high degree of Denisovan ancestry in Tibetans, suggesting that this population acquired advantageous alleles for high altitude life through ancient admixture [75,76,77]. In contrast to these evidences of positive selection, evidence for balancing selection in humans is largely circumstantial [78]. However, host defense genes such as those encoding several membrane glycoproteins, the *KIR* regions that coevolve with *HLA* ligands, and other genes encoding proteins involved in cell migration or innate immunity, apparently are subject to this otherwise rare selective regime [79,80,81,82,83]. The *HLA* region, a paradigm of balancing selection in humans, harbors functional variants that were probably introgressed from Neanderthals and Denisovans [84]. An alternative explanation by Yasukochi and Ohashi [85] based on phylogenetic analysis does not support the introgression hypothesis and concludes that it is highly likely that the supposedly introgressed allelic lineage HLA-B*73 has been maintained in the direct ancestors of modern humans [85].

Increasing evidence suggests that regulatory variants play a central role in adaptive processes [86,87,88]. A compelling example of local adaptation detected on the expression level is at the apelin receptor gene *APLNR*. Apelin is a signal peptide that influences several aspects of cardiac, digestive, brain, and vascular function, including regulation of oxygen levels. This gene exhibits strong allele-specific expression favoring the Neanderthal allele in brain tissues, but allele-specific expression favoring the modern human allele in non-brain tissues [55]. There are also a number of examples of local adaptation driven by regulatory variants resulting in population differences in immune responses [71,72,89]. Despite the evidence that functional archaic alleles (non-synonymous and associated with expression) have decreased in frequency over the past 8500 years, four loci were identified where the archaic alleles associated with differential expression show large increases in frequency over time. Among these are introgressed alleles modifying expression of the *OAS1*/*OAS2*/*OAS3* genes involved in innate immunity and whose tissue-specific effects suggest that they may be functionally relevant [71,89]. Archaic alleles in *OAS1* are associated with higher expression in subcutaneous adipose tissue and sun-exposed skin, while higher expression of *OAS2* in the thyroid and *OAS3* in the pancreas and vagina is associated with archaic alleles. In contrast, individuals carrying archaic alleles show down-regulation of *OAS1* in esophagus mucosa and spleen, *OAS2* in fibroblasts, and *OAS3* in fibroblasts as well as in esophagus mucosa, spleen and three brain regions (hippocampus, putamen, and caudate nucleus) [84]. Other examples of local adaptation influencing the levels of expression include expression of gene *ERAP2*, involved in susceptibility to Crohn’s disease; *CCR1*, limiting leukocyte recruitment and preventing inflammatory responses; *HLA-DQA1*, associated with susceptibility to celiac disease; and *TLR1*, associated with markedly lower levels of inflammatory response gene expression [71,72]. Apparently, introgression from Neanderthals also contributed to the diversification of transcriptional responses to infection in human populations. The introgressed genetic segments in European genomes contain regulatory variants with effects on steady-state expression and responses to *TLR7*/*8* stimulation and influenza virus [72,78]. Furthermore, the archaic variants of several expression quantitative trait loci (eQTLs) have been reported as potential candidates for adaptive introgression conferring better adaptation through the regulation of gene expression. Examples are the gene *DARS* associated with neuroinflammatory and white matter disorders [71]; the archaic variants of *OAS* locus apparently associated with diverse flavivirus resistance phenotypes [90]; and *PNMA1* harboring a response eQTL for influenza virus and stimulating interferon production [72]. Another regulatory archaic variant that modifies the expression of *TNPO3* in the brain is associated with multiple autoimmune phenotypes [83]. All these studies clearly show that selection and archaic admixture affect substantially present-day inter-population differences in immune responses, at least in terms of transcriptional variability, supporting the notion that variation in gene expression has been an important vehicle for human adaptation [86]. Furthermore, it has been shown that the higher frequency archaic variants contribute significantly more to gene expression changes than lower frequency archaic variants. This suggests that at least some of the archaic alleles that modify gene expression may have been driven to higher frequencies in many human populations by positive selection, supporting the idea that changes in gene expression are likely to have important adaptive effects in humans [89].

However, whatever the potential benefits of archaic introgression in the past, alleles of Neanderthal origin have been also associated with several neurological, dermatological, and immunological phenotypes, indicating an influence of ancient admixture on current disease risk in humans [91,92,93]. For example, introgressed alleles associated with the immune system response can increase the risk of inflammation or autoimmunity under environmental factors changing overtime [94,95,96,97,98]. The case of celiac disease neatly illustrates the tradeoff between past selection and current maladaptation. Taskent et al. [93] detected evidence of Neanderthal introgression in the chemokine receptor (*CCR*) gene family constituting the risk alleles for celiac disease, which was possibly maintained by selective forces in early European population. Furthermore, population genetic analyses have shown that the high frequency of several risk alleles of genes associated with celiac disease such as *IL12A*, *IL18RAP* and *SH2B3* [99] in Europeans results from past positive selection events [94,100]. Another example comes from a nonsynonymous variant of the *ZNF365D* gene present in ~32% of Europeans and absent from Africans, which was inherited from Neanderthals and is associated with a higher risk of Crohn’s disease [36]. Likewise, variants of gene cluster *TLR6-1-10* inherited from Neanderthals and Denisovans and present in Europeans and Asians has been associated with greater susceptibility to allergies [101].

Further investigations are required, but the studies published to date have provided invaluable resources and increased our understanding of the molecular and cellular processes underlined by introgressed genetic variants and different selective regimes acting on them.

## 4. Conclusions and Perspectives

The ongoing deluge of sequencing data from thousands of individuals and different populations worldwide, including some archaic hominins and ancient AMH genomes, has provided new insights into the evolutionary history of our species. Genomic studies of introgression between early Eurasians and archaic human species, such as Neanderthals and Denisovans, are beginning to offer novel insights into the evolutionary and phenotypical consequences of hybridization. There is quite common evidence for widespread selection against introgression across the genome, but adaptive introgression may also be considered an important force driving adaptation of modern humans to new environments. However, additional human dataset advances, integration of different sources of information and development of new statistical and analytical methods are critical for understanding the biological and medical implications of such signals of selection.

Table S1 illustrates that our knowledge of the functional consequences of the introgressed variation is essentially based on populations with European ancestry. Informative data from other ethnic groups and sequencing additional samples from ancient hominins will further deepen our knowledge of the contribution of archaic hominins to the diversity of human traits and complex diseases. Furthermore, it will help to identify the functional changes that have contributed to human adaptation and survival over time. Moreover, multigenerational prospective cohort studies from multiple human populations will allow direct measurements of genetic variation and selection intensity for common traits in contemporary populations, performed in a range of nutritional, cultural and geographic conditions, constituting the best way of characterization of the magnitude and importance of complex ecological, epidemiological, demographic and evolutionary shifts. In addition to the genome databases of European origins as the Framingham cohort in the USA, the Uppsala Birth Cohort in Sweden and the Lifelines Cohort in the Netherlands such cohorts now include the H3Africa Initiative on Human Heredity and Health in Africa and the Tohoku Medical Megabank Project in Japan. Data desideratum supplemented with genomic and medical information will further increase our understanding of the antagonistic pleiotropic effects that contribute to the burden of non-infectious diseases and provide new clues to disease causes, potential therapies and possible adverse effects of novel therapies [96].

In the case of integrating different sources of information, studies of genetic variants with regulatory effects on gene expression (eQTL) have already provided insight into the genetic and evolutionary determinants of population phenotypic diversity [102]. One of the first global approaches is the Genotype-Tissue Expression (GTEx) project. GTEx explores the landscape of gene expression across 54 different tissues, providing the richest catalog of tissue-specific and shared eQTL [103,104]. It was already used in the analyses of expression patterns of introgressed haplotypes in the recent studies conducted by McCoy et al. [55] and Dannemann et al. [89]. The future population genetic analyses should extend this across-tissue rationale to multiple populations from different ethnic backgrounds to provide a comprehensive picture of the physiological mechanisms underlying adaptation to environmental pressure and the maintenance of homeostasis.

In the case of new methods, the challenge for the future will be to develop robust statistical models and computational methods for detecting selection, quantifying the frequency of adaptive introgression more widely and understanding the circumstances where it is likely to play a predominant role in adaptation. An exciting future prospect is that our interpretations of observations in nature will be aided by simulation studies [33] and empirical studies of the consequences of introgression for phenotype and fitness [55]. In this context, there are no accurate estimates of the timing of most of the signatures of selection now being detected in the human genome (lactase persistence as an exception) [105,106], or good methods for estimating the ages and natures of the environments in which past selection occurred [97]. Future studies should be able to discriminate with confidence between different time-scales of selection, for example, as a result of the agricultural revolution 8000–10,000 years ago or the industrial revolution 100–300 years ago.

In conclusion, the integration of all of these datasets into a clinical, epidemiological, and population genetics framework will provide new insights on the history of adaptations in the genus *Homo*, and the ways our genetic and non-genetic makeup, together with changes in our environment and cultural behaviors, influence phenotypic variation in both health and disease.

## Supplementary Materials

The following are available online at http://www.mdpi.com/2073-4425/9/7/358/s1, Table S1: Phenotypic implications of putatively introgressed genomic regions.
